# 127 Anxiety Disorder Symptomology Found to Be Prevalent in Burn-injured Youth

**DOI:** 10.1093/jbcr/irac012.129

**Published:** 2022-03-23

**Authors:** Ruth B Brubaker Rimmer, R C C Bay, Emile T Kalil, Daniel W Chacon, Kevin N Foster

**Affiliations:** Arizona Burn Center - Valleywise Health, Phoenix, Arizona; A. T. Still University, Mesa, Arizona; Mid-Atlantic Burn Camp, Highland, Maryland; Alisa Ann Ruch Burn Foundation, SAN FRANCISCO, California; The Arizona Burn Center Valleywise Health, Phoenix, Arizona

## Abstract

**Introduction:**

Anxiety Disorder (AD) is common in inpatient pediatric burn patients and likely related to pain/stress associated with acute care. This study ascertained if burn survivors reported higher anxiety levels based on sex, visibility of scars, or TBSA ≥ 50%.

**Methods:**

Burn-injured youth completed the Screen for Child Anxiety Related Disorders (SCARED) with parental consent. This 41 item self-report measures DSM-IV pediatric anxiety disorder symptoms: panic disorder (PD), separation anxiety (SA), generalized anxiety disorder (GAD), social phobia (SP) school phobia (SCP) and total anxiety (TA). The percentage of respondents above threshold for each disorder was calculated.

**Results:**

112 survivors, mean age of 13, included boys (51%) & girls (49%). 83 reported visible scars. Females had higher percentages for TA (53%) vs. males (21%) (p < 0.001), PD (47%) vs. (7%) (p< 0.001), GAD (40%) vs. (16%) (p < 0.005), & SA (51%) vs. (21%) (p < 0.001). Youth with TBSA ≥ 50% (n=22) had higher precents for GAD (46%) vs. < 50% (24%) (p < 0.01). The visibly scarred had higher percent for GAD (38%) vs. hidden (7%) (p< .01).

**Conclusions:**

Female, visibly scarred, and patients with burns > 50% revealed increased AD symptoms. AD may be chronic, interfere with a child’s home & school function and lead to chronic distress, substance abuse, and isolation. Screening for anxiety in burn-injured youth is recommended.

